# A Quantitative Model of Transcriptional Regulation Reveals the Influence of Binding Location on Expression

**DOI:** 10.1371/journal.pcbi.1000773

**Published:** 2010-04-29

**Authors:** Kenzie D. MacIsaac, Kinyui A. Lo, William Gordon, Shmulik Motola, Tali Mazor, Ernest Fraenkel

**Affiliations:** 1 Department of Electrical Engineering and Computer Science, Massachusetts Institute of Technology, Cambridge, Massachusetts, United States of America; 2 Department of Biological Engineering, Massachusetts Institute of Technology, Cambridge, Massachusetts, United States of America; 3 Computer Science and Artificial Intelligence Laboratory, Massachusetts Institute of Technology, Cambridge, Massachusetts, United States of America; Weizmann Institute of Science, Israel

## Abstract

Understanding the mechanistic basis of transcriptional regulation has been a central focus of molecular biology since its inception. New high-throughput chromatin immunoprecipitation experiments have revealed that most regulatory proteins bind thousands of sites in mammalian genomes. However, the functional significance of these binding sites remains unclear. We present a quantitative model of transcriptional regulation that suggests the contribution of each binding site to tissue-specific gene expression depends strongly on its position relative to the transcription start site. For three cell types, we show that, by considering binding position, it is possible to predict relative expression levels between cell types with an accuracy approaching the level of agreement between different *experimental* platforms. Our model suggests that, for the transcription factors profiled in these cell types, a regulatory site's influence on expression falls off almost linearly with distance from the transcription start site in a 10 kilobase range. Binding to both evolutionarily conserved and non-conserved sequences contributes significantly to transcriptional regulation. Our approach also reveals the quantitative, tissue-specific role of individual proteins in activating or repressing transcription. These results suggest that regulator binding position plays a previously unappreciated role in influencing expression and blurs the classical distinction between proximal promoter and distal binding events.

## Author Summary

Gene expression is controlled, in large part, by regulatory proteins called transcription factors that bind specific sites in the genome. A major focus of molecular biology has been understanding how these transcription factors interact with the cell's transcriptional machinery, the genome, and with each other to turn genes' expression on and off in various physiological contexts. Previous approaches for modeling transcriptional regulation have focused on the complex combinatorial interactions between groups of transcription factors at regulatory sites, or on the specific activating or repressive functions of individual proteins. In this work, we present a new modeling framework and demonstrate that an equally important, and previously overlooked, consideration in predicting the effect that a regulatory site has on gene expression is simply its location relative to the transcription start site of nearby genes. Our results show that, in general, the closer a binding event is to a gene's transcription start site, the more it influences expression. We also show that considering the particular proteins bound at a regulatory site helps predict the expression of nearby genes. However, considering the sequence conservation level of these sites does not lead to more accurate predictions.

## Introduction

Control of gene expression programs across diverse tissues and developmental stages is achieved through networks of proteins interacting with specific regulatory sites in the genome. Pioneering work on several individual promoters, including those of beta-interferon [1] and *endo16* [2] have revealed that the relationship between binding events and transcriptional outcomes can be quite complex. The advent of chromatin immunoprecipitation (ChIP) coupled with high throughput microarray (ChIP-chip) or sequencing (ChIP-seq) technology has highlighted the challenges of understanding transcriptional regulation. These technologies have been used to map binding sites on a genome-wide scale [3], [4], [5], [6], [7], [8], [9], [10], and have shown that regulatory proteins typically bind thousands of genes. As might be expected, given the importance of combinatorial control in well-studied promoters, only a subset of the detected regulator binding sites are functional, while many binding events play no direct role in determining transcription levels [11]. A further complication arises from the observation that distal enhancers, which can be located many kilobases from transcription start sites, can be important drivers of expression [12], [13] thereby vastly increasing the number of binding events that must be considered potentially functional for each gene. Moreover, it is usually unclear which binding events regulate which genes. In this study, we present a new model of transcriptional regulation that addresses these key challenges. We identify sites of combinatorial control by performing high throughput ChIP experiments on p300, CREB-binding protein (CBP), the deacetylase SIRT1 and on multiple DNA-binding transcription factors in three different tissues. We then develop a simple framework that predicts the quantitative effect of binding on gene expression and reveals the relative contributions of each protein to the combinatorial control of transcription. Remarkably, we find that the effect a regulatory site has on a gene's expression is, to a large extent, dependent on its proximity to the gene's transcription start site.The model predicts that both conserved and non-conserved sites have important roles in determining transcription outcomes. Further, we find that the data better support a model where individual regulatory sites affect the expression of multiple nearby genes than a model where these sites regulate only the most proximal gene.

## Results

### Identification of regulatory regions

We identified sites of combinatorial control by performing ChIP on samples from mouse liver and 3T3-L1 cells using an antibody specific to p300, which has been used similarly in previous studies [8], [9], as well as antibodies for several proteins with transcriptional activation function in these cell types (Table 1) and by analyzing previously published data for PPARγ and RXR in 3T3-L1 cells [14]. Immunoprecipitated DNA was sequenced, the 35bp reads were aligned to the reference mouse genome, and regions with significant levels of binding relative to a set of control reads were identified. We also performed ChIP-chip experiments in liver and cerebellum using an antibody specific to CBP, a transcriptional coregulator closely related to p300, using promoter microarrays.

**Table 1:** Anti-sera used in ChIP experiments.

Protein	Antibody	Source	Cell types
**CBP**	sc-369X	Santa Cruz	liver, cerebellum
**C/EBPα**	sc-9314X	Santa Cruz	liver, 3T3-L1
**E2F4**	sc-1082X	Santa Cruz	liver, 3T3-L1
**FOXA1**	ab5089	Abcam	liver
**FOXA2**	sc-6554	Santa Cruz	liver
**p300**	sc-585	Santa Cruz	liver, 3T3-L1
**pCREB**	sc-7978X	Santa Cruz	liver
**Sirt1**	sc-19857	Santa Cruz	cerebellum

**Figure 1:**
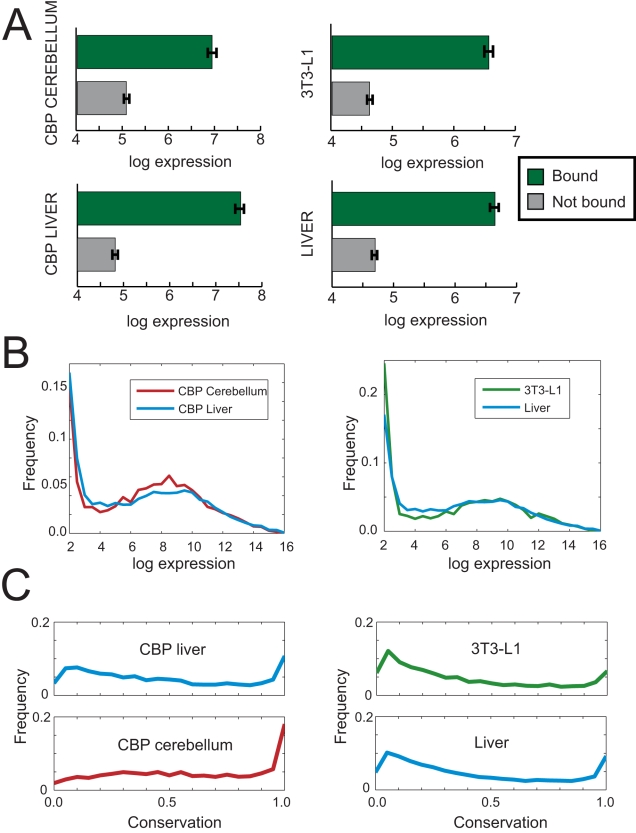
Characteristics of bound genes and bound regions. Putative regulatory regions in liver and 3T3-L1 cells were identified from ChIP-seq experiments and are defined as sites bound by p300 and/or at least two other transcription factors. We performed a similar analysis in liver and cerebellum using ChIP-chip with promoter microarrays, where a regulatory region is defined as any site bound by CBP. (A) Genes with a regulatory region within 5kb of their transcription start site have a higher mean expression level than genes with no binding event. Error bars indicate +/− s.e.m. (B) Bound genes display large variation in levels of absolute gene expression. (C) Putative regulatory regions show great variation in their sequence conservation levels. Conservation level was calculated as the maximum 100bp moving average of Phastcons scores from alignments of placental mammal genomes.

**Figure 2:**
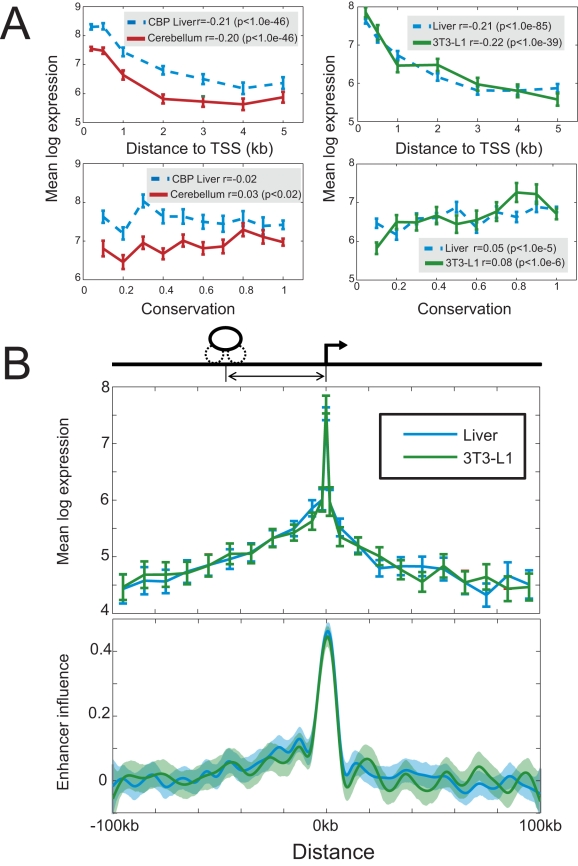
Binding site position, but not sequence conservation, is strongly associated with gene expression level. (A) The mean log expression of bound genes is shown in each tissue as a function of both the distance between the transcription start site and the nearest regulatory region identified by ChIP, and the maximum conservation score of any regulatory region within 5kb of that gene's TSS. Error bars indicate +/− s.e.m. Also shown is the Spearman correlation, and associated p-value from a right-tailed t-test, between log expression and the distance and conservation measures. (B) In the upper plot the mean log expression of genes in liver and 3T3-L1 cells is shown as a function of the location of the nearest binding site over a 200kb window. Error bars indicate +/− s.e.m. In the lower plot we show the influence function, which measures a binding event's predicted effect on expression as a function of position, obtained by fitting our predictive model to 1,000 bootstrapped samples of ChIP and expression data in each tissue. Shaded regions show the empirical 99% confidence intervals obtained from the bootstrap iterations.

**Figure 3:**
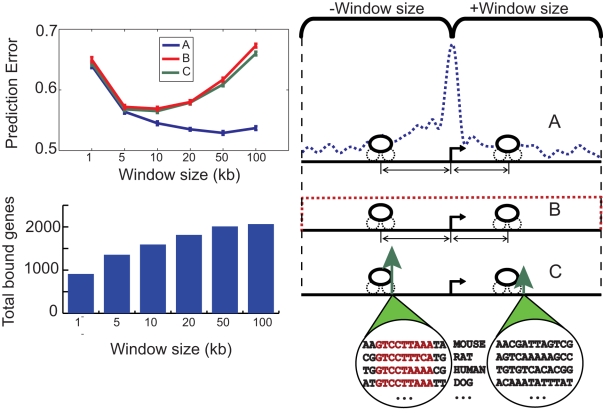
Regulatory region position predicts tissue-specific expression. We compared three models relating regulator binding to relative transcription levels in liver and 3T3-L1 cells. The first model (A) learns a binding site's expression influence based on its location relative to the TSS. The second model (B) treats all binding events equally regardless of position. The third model (C) learns a binding site's expression influence based on its level of sequence conservation. We tested each model using several different distance cutoffs to identify bound genes. The bar graph shows how the number of genes included in the analysis increases as this cutoff is increased. Each model's performance, as measured by mean-squared prediction error on held out test data, is shown as a function of distance cutoff. Error bars indicate +/− s.e.m. The model that learns influence from position significantly outperforms the other approaches.

The ChIP-seq analysis identified 22,191 and 7,821 sites bound by p300 or at least two other regulators (which we will refer to as putative regulatory regions) in liver and 3T3-L1 cells respectively (see Methods). The vast majority of these sites occur within 100kb of known genes but most are located outside of the proximal promoter (Figures 1 and 2 in Text S1): 92% of regulatory sites in liver and 93% in 3T3-L1 cells occur outside the 500bp window centered on each transcript's transcription start site (TSS). The ChIP-chip promoter array experiments revealed 3,326 and 3,187 CBP-bound regions in liver and cerebellum; 70% of these sites in liver and 51% in cerebellum occur outside the proximal promoter. Several sites directly overlap previously characterized transcriptional enhancers [15], [16], [17], [18], [19], [20], [21] (Figure 3 in Text S1).

### Binding proximity predicts transcription

Understanding the relationship between regulator binding and transcription is a complicated task. We find that binding within 5 kilobases (kb) of a gene's transcription start site (TSS) is associated with higher transcript levels (Figure 1A), however it provides limited information about the magnitude of tissue-specific transcription levels. Bound genes display a wide range of expression values (Figure 1B). This variation may be explained, in part, by the action of distal regulatory sites located further than 5kb from the gene. However, as we begin to consider binding events further from the TSS the situation becomes increasingly complicated as more, potentially non-functional, binding sites become associated with each gene. It is also difficult to associate binding events with the genes they regulate. For example, approximately 41% of regulatory sites identified in liver and 45% in 3T3-L1 cells are located within 50 kb of the TSS of two or more genes.

The problem of identifying functional regulatory regions has been addressed using sequence conservation [22], [23]. We found that bound regions vary significantly in their degree of sequence conservation (Figure 1C) and wished to explore whether more highly conserved sites were more likely to be functional. When we examined the mean expression level of genes in each tissue as a function of the conservation level of nearby binding events, we found a weak or non-existent relationship (Figure 2A).

**Figure 4:**
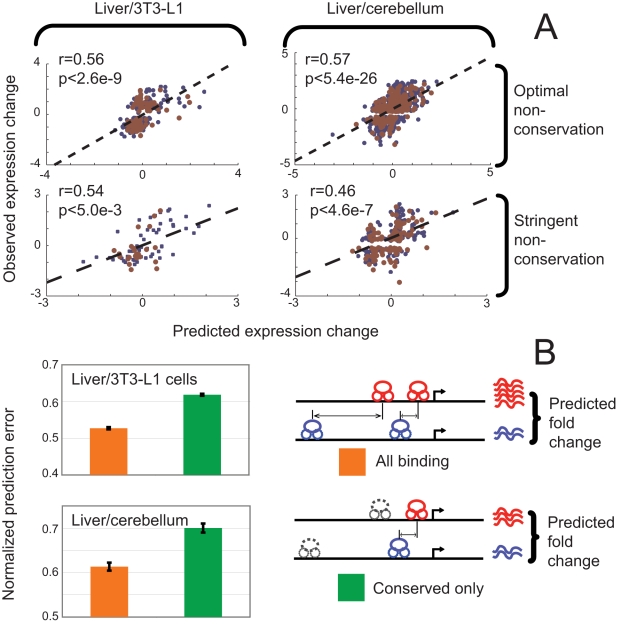
Non-conserved binding events predict expression. (A) Scatter plots of observed and predicted expression difference are presented for differentially expressed genes bound only at non-conserved regions at stringent and moderate conservation thresholds. Training data points are shown in blue and test data is shown in red. Each non-conserved binding event's effect on transcription was modulated by its distance to the TSS. In both tissue pairs, and at both conservation thresholds, the model's predictions are strongly correlated with observed expression differences. (B) The expression difference of genes bound at both conserved and non-conserved sites was predicted using only conserved sites, and the prediction error was compared to that obtained when both conserved and non-conserved binding sites were used. Including non-conserved regions significantly improved performance in both tissue pairs. Error bars indicate +/− s.e.m.

Previous computational models of transcriptional regulation have frequently ignored the effect of distance between a binding site and a gene [24], [25], [26] or have considered location only for the purposes of detecting positional binding preferences of proteins in the proximal promoter [27], [28]. Previous approaches have also not accounted for the effect of very distal binding sites on expression. Interestingly, we find that transcription levels are correlated with the proximity between a gene's TSS and the closest bound region (Figure 2A), and that this statistical relationship persists over tens of kilobases (Figure 2B). Surprisingly, this relationship is even observed at a distance resolution of hundreds of nucleotides within the proximal promoter (Figure 4 in Text S1). To further understand the relationship between expression and regulator binding location we developed a simple quantitative model that predicts transcription level as a function of transcription factor binding position. We assume that the mean expression level of a gene is determined by contributions from all individual regulatory sites in the vicinity of that gene, and that each regulatory site may regulate the expression of multiple genes. The functional relevance of a region depends on its position relative to the TSS; this relationship takes the form of an influence function that is fit to the data during model training. This approach allows proximal sites to be treated differently than distal sites, or upstream and downstream sites to be treated differently. We first used our model to predict the absolute expression levels of genes in liver and 3T3-L1 cells from the location of p300 and clustered transcription factor binding sites. We considered all binding events located within 100kb of each gene's TSS. The correlation between predicted and observed transcript abundance in held-out test data is highly statistically significant (Table 1 in Text S1). Notably, the predicted relationship between position and expression influence is nearly identical in both cell types (Figure 2B). The influence of an enhancer falls off approximately linearly as the position moves further away from the TSS. Sites located within approximately 10kb of the TSS are statistically associated with the highest transcription levels, and regulatory regions located upstream of the TSS are predicted to have a somewhat greater effect on transcription than downstream events. Although proximal sites have the greatest influence, binding sites located up to 50kb away from the TSS are predicted to have a significant effect on transcription, consistent with previous observations that enhancers may act at very long distances to affect expression [12], [13].

### Predicting differential expression level

If the location of a regulatory site does, in fact, have a large effect on gene expression, then *changes* in gene expression between cell types should be associated with *changes* in the location of binding sites. To examine this question, we used all the liver and 3T3-L1 binding events identified in ChIP-seq experiments to predict relative expression of differentially expressed genes in these cell types. We find that regulatory sites located within 10kb of differentially expressed genes are more likely to be unique to a single tissue than those in the vicinity of non-differentially expressed genes (Table 2 in Text S1). Genes that exhibit no difference in expression are also much less likely to be bound than differentially expressed genes: 7,628 of 15,568 non-changing genes had no putative regulatory site within 10kb of their TSS, compared to only 417 of 2,124 differentially expressed genes. In order to evaluate the importance of binding site position in predicting the functional relevance, we compared our model's performance to two competing models: one that weighted binding events equally regardless of position (as was done in all previously published studies), and a second that weighted the contributions of bound regions by sequence conservation, allowing highly conserved regulatory regions to be weighted differently than regions with low conservation. We fit each model using two-thirds of the bound, differentially expressed genes, and evaluated their ability to predict the magnitude of expression differences for the remaining third of the genes, repeating this process 100 times using randomly sampled test and training data.

The position-based model of transcription produces significantly more accurate predictions than the uniform weighting and the conservation-based approaches (Figure 3). To evaluate the importance of distal binding events in predicting expression, we identified bound genes using several distance cutoffs, ranging from the 1kb proximal promoter to a distance of 100kb from the gene's TSS. The position-based model out-performs the other models across a wide range of distance windows. At the 100kb cutoff, 2,205 of the 2,309 differentially expressed genes identified are bound in at least one tissue (Figure 3). Even when including these very distal sites in the analysis, many of which are presumably non-functional, our predictions have a median correlation of 0.69 with observed expression levels of held-out test genes compared to 0.58 for the conservation-based model and 0.57 for the model that weights binding events uniformly. This value approaches the correlation level observed for relative expression measurements made using different experimental platforms [29], [30] and indicates that regulatory site position has a substantial effect on transcription levels in these. As a further control, we performed an additional 100 bootstrap trials with randomly permuted expression values across differentially expressed genes. In these trials, our model's prediction accuracy was statistically no better than a strategy of predicting the mean expression value in the training set.

Including binding events up to 50kb away from the TSS improves expression predictions, demonstrating the importance of these distal sites. However, weighting the influence of each regulatory region appropriately is crucial; the models that do not consider position both show a drastic deterioration in prediction accuracy as the distance cutoff increases. Interestingly, the simple uniform weighting model performs about as well as the model that weights sites by sequence conservation, indicating that conservation is of limited use in identifying functional binding events from ChIP data.

To address whether these data support the hypothesis that individual regulatory sites regulate multiple genes, we compared the prediction accuracy of our model to one where regulatory sites are assumed to regulate expression of only the closest transcript. We first associated binding events in liver and 3T3-L1 cells to transcripts, assuming they regulate only the nearest gene. We then trained our position-based transcriptional model and predicted the expression of held-out genes. These predictions were compared to those obtained, for the same set of genes, without the constraint that a site regulates a single gene. The difference in prediction accuracy is dramatic. The mean-squared prediction error over 100 bootstrapped trials was 0.73+/0.03 s.d. when we assume that binding events regulate only the closest gene. This improved by approximately 8 standard deviations to 0.48+/−0.02 s.d. for a model where binding events may regulate many genes.

### Binding at regions with low sequence conservation is functional

**Figure 5:**
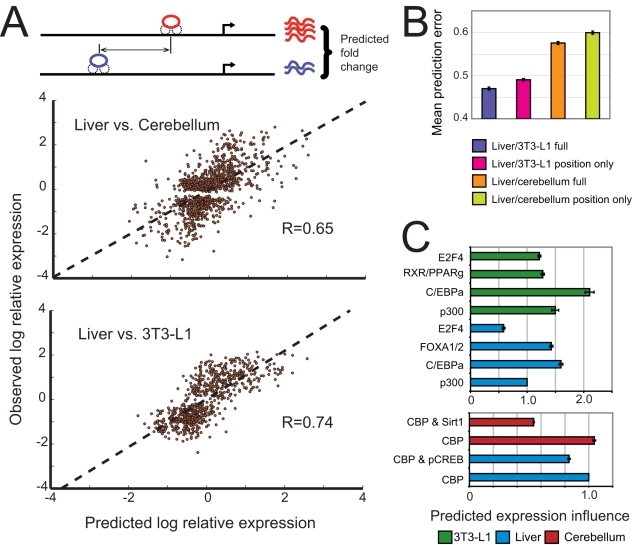
Transcriptional regulators have distinct influences on expression. (A) Shown are representative scatter plots of predicted vs. observed expression differences for held out test genes in liver/cerebellum and liver/3T3-L1 cells. Predictions were made using a transcriptional model that takes into account the influence of both the genomic position and the particular proteins bound by a site. The median correlation from 11 separate trials was 0.65 and 0.74 for liver/cerebellum and liver/3T3-L1 respectively. (B) The prediction error of the full model that includes individual transcription factor influence weights is compared to a model that uses only position to predict influence. Modeling the influence of bound regulators improves predictive performance. Error bars indicate +/− s.e.m. (C) The expression influence for each protein is learned in our transcriptional model. Sites bound by proteins with known repressive activity (E2F4 and Sirt1) are predicted to have the smallest influence.

To further explore the role of non-conserved regulatory sites we identified bound regions in each tissue that showed low sequence conservation levels, using the conservation threshold that best distinguished bound regulatory regions from randomly selected DNA sequences (Figure 5 in Text S1). At this threshold, approximately 59% of sites from ChIP-seq experiments in liver and 47% in 3T3-L1 cells are non-conserved. Similarly, 44% of CBP sites in liver and 28% of sites in cerebellum are non-conserved. Genes located within 5kb of these sites in our experiments were associated with high levels of gene expression (Figure 6 in Text S1). Next we identified 261 differentially expressed genes in liver and 3T3-L1 cells bound (within 50kb) at *only* non-conserved regions. In a similar fashion, we identified 884 differentially expressed genes bound only at non-conserved regions by CBP in liver and cerebellum. We performed the training and test procedure described above and determined whether the locations of these non-conserved sites predicted gene expression (Figure 4A). In both liver/3T3-L1 cells and in liver/cerebellum the position of non-conserved binding is a strong predictor of relative expression level. Our predictions have a mean correlation of 0.56 with observed expression values in liver/3T3-L1, significant at p<2.6e-9 by a right-tailed t-test. In liver/cerebellum the mean correlation is 0.57, significant at p<5.4e-26. We then repeated the analysis using an even more stringent conservation threshold (see Methods) and found that non-conserved sites were still highly predictive of expression (Figure 4A). We also examined genes bound at both conserved and non-conserved sites within 100kb of their TSS and asked whether the conserved sites alone were adequate to predict expression. We first predicted expression using only conserved sites and then repeated the analysis using all bound regions. Underlining the importance of non-conserved regulatory regions, we find that considering both the conserved and non-conserved sites results in significantly more accurate predictions (Figure 4B).

### Revealing the role of specific regulators

Although binding site position is very important in determining expression influence, the function of a regulatory region is also determined by the particular transcription factors that bind to it. We therefore extended our transcriptional model so that the relevance of any particular regulatory site was determined by both its location and the particular regulators that were bound. Each protein's effect on transcription was estimated by including a protein-specific weight that modulated the expression influence of the site. We tested this approach on ChIP-seq and expression data in liver and 3T3-L1 cells, including binding data for an additional regulator, E2F4, in each tissue. We estimated the influence of p300, C/EBPα, FOXA1/A2, and E2F4 in liver, and p300, C/EBPα, PPARγ/RXR, and E2F4 in 3T3-L1 cells. In total, 2,038 differentially expressed genes were analyzed. Our predictions have a median correlation of 0.74 with observed expression differences on held out test data, ranging between 0.72 and 0.76 in 11 separate trials (Figure 5). Our simple predictive framework remarkably accounts for over 50% of the variance in observed relative expression levels, and gives better predictions than a model that considers only binding site position. The influence learned for each protein provides evidence of its function in these cell types. For example, C/EBPα is associated with the strongest activation in both cell types, in agreement with its well-characterized role in these cell types [31]. In contrast E2F4 is associated with the lowest levels of activation in both cell types; its influence weight of 0.52 in liver indicates that it actually attenuates an enhancer's effect on expression in this tissue, consistent with its previously described transcriptional repressor activity [32]. We performed a similar analysis in liver and cerebellum by collecting ChIP-seq data for the histone deacetylase Sirt1 in cerebellum, and ChIP-chip data for the transcription factor pCREB in liver. Modeling the different transcriptional influences of CBP sites that are also bound by pCREB or Sirt1 resulted in more accurate expression predictions. The median correlation between observed and predicted expression difference in liver and cerebellum was 0.65, ranging between 0.62 and 0.68 over 11 separate trials. Sirt1 has the opposite enzymatic activity to CBP/p300, and is known to repress p300 activation of transcription in certain contexts [33]. As expected, sites in cerebellum that are bound by Sirt1 have only about half as much influence on expression levels as CBP sites that do not recruit Sirt1. In a separate analysis, we modeled the effect of CBP binding affinity on expression influence, up weighting sites with higher ChIP enrichment ratios (Text S2). Accounting for the effect of binding affinity results in a very significant 15.5+/−1.3% mean improvement in prediction accuracy over ten separate trials.

## Discussion

In this study, we address a central problem in the study of transcriptional regulation by developing a model that reveals the function of transcription factor binding sites in terms of their genomic position and the particular regulators they bind. Experimental approaches combining ChIP with microarray and sequencing technologies have led to tremendous progress in mapping transcriptional regulatory sites across the genome. However, progress in determining the function of these sites has been slower. In part this is because static maps of regulator binding give an incomplete picture of the complexity that arises from dynamic signaling and binding events, but progress has also been slowed by the absence of a simple framework that links regulatory network architecture (as defined by the location of regulatory regions in the genome) to transcription.

To understand the functional role of these regulatory sites, we developed a simple model that accurately predicts the expression difference between cell types based only on binding site positions. The correlation of the predictions with measured values approaches the correlation observed between different *experimental* platforms and can remarkably explain over half the variance in the relative transcription levels of differentially expressed genes.

Previous work has suggested that functional transcription factor binding sites tend to cluster near the transcription start site (TSS) of the genes they regulate [34], [35], [36]. Our results agree with these observations; binding events that are very close to the transcription start site are predicted to have a disproportionately large effect on expression. However, many genes show large differences in tissue-specific expression that are apparently driven by much more remote events as evidenced by our ability to predict these differences even when no binding event is detected within 1kb of the TSS (Figure 7 in Text S1). For the proteins and tissues analyzed in this study, a regulatory site's position relative to a gene's transcription start site appears to be an extremely important determinant of its effect on that gene's expression. Although we are aware of an *in vitro* study where a falloff in transcription rate was observed as a regulatory site's location was moved further from the TATA box over a range of approximately 100bp in a series of reporter constructs [37], to our knowledge the intriguing effect of position has not been previously reported as a general feature of transcriptional regulation in an *in vivo* system.

Interestingly, our analysis supports a model where binding events frequently regulate the expression of multiple genes over one where bound regulators affect the expression of only the most proximal gene. Based on our observation that binding sites located within 50kb of a gene significantly influence its expression level, we estimate that approximately 40–45% of regulatory sites may affect the expression of more than one transcript.

In contrast to the strong relationship between the location of binding and transcription, there is little relationship between sequence conservation and expression. Including binding to non-conserved sequences in our models improves their accuracy significantly over models built using only binding to conserved sequences. Previously we, and others, have shown that the sites targeted by individual DNA-binding proteins can vary across species even when tissue-specific gene expression is conserved [38], [39]. Taken together, these findings suggest that organisms can achieve similar gene expression patterns through diverse mechanisms. Because transcription integrates binding events that are distributed over great distances, there is a reasonable probability that the evolutionary gain or loss of regulatory regions at one locus can be compensated for by mutations at other sites. More work is needed to determine whether the quantitative relationship between binding and expression is similar across mammals.

Regulatory sites have been classically divided into promoter-proximal elements, which are within approximately 200 base pairs of the start site, and enhancer elements [40]. Surprisingly, we find an almost linear decrease in the effect of a regulatory site over a region of many kilobases, encompassing both proximal promoters and distal enhancers. Our results suggest that a more critical distinction may be between those binding events within or beyond 50 kilobases and that the net transcription level of a gene is the result of integrating a potentially large number of binding events.

The results presented here represent a significant step towards a quantitative framework for understanding gene expression. The statistical relationship between enhancer position and transcription level is clear, and this observation should lead to more accurate models of transcriptional regulation. However, many other factors have a profound effect on enhancer function including which coregulators are recruited, the nuclear concentrations of transcription factors, binding of small molecules that modulate enzymatic activities and interaction surfaces, and any signaling events leading to post-translational modification of regulators. In addition, it is possible that different types of enhancers exist that vary in the relationship between enhancer position and transcription level. Enriching the modeling framework presented here by incorporating additional types of data that address these questions (e.g. CTCF enhancer binding sites) may lead to a greater understanding of regulatory networks and their relationship to developmental and disease processes.

## Methods

### Chromatin immunoprecipitations

Male C57BL/6J mice were purchased from Taconic. Animals were provided with water and chow without restriction. Hepatocytes were harvested by direct perfusion of the liver in anaesthetized animals using PBS, followed by crosslinking with a 1% formaldehyde solution. The liver was then removed and crosslinked for another 10 minutes followed by neutralization with glycine. This cellular material was homogenized, washed and passed through a sucrose gradient to enrich for hepatocytes. These were rinsed with 1× PBS, pelleted, and either used directly in ChIP experiments, or frozen in liquid nitrogen for later use. Mouse cerebella were harvested from male C57BL/6J mice and crosslinked, homogenized, and neutralized in a similar manner. Murine preadipocyte 3T3-L1 cells were induced to differentiate to mature adipocytes using a standard protocol [41] cross linked for ten minutes and then quenched with glycine. ChIP experiments were performed as previously described [6], [42] using antisera listed in Table 1.

### Processing of ChIP data

ChIP-seq analysis of immunoprecipitated DNA was carried out using the standard Illumina protocols and analysis pipeline. The enrichment of genomic regions for protein binding was assessed relative to a set of control reads obtained by sequencing unenriched whole-genome DNA. Bound regions were identified using the MACS algorithm [43] with a calculated alignable genome size of 2.107 Gbp [9] and an enrichment p-value cutoff of 1e-6.

After scanning, ChIP-chip data from Agilent proximal promoter arrays were analyzed using the Redwing algorithm. Redwing extends a previously presented analysis framework [44] and is detailed in Text S2. Binding scores were obtained by convolving Redwing's binding estimates with a 400bp rectangular window. These smoothed binding scores were compared to scores obtained by analyzing randomly permuted probe intensity data for each experiment. Scores with an estimated FDR of < = 0.05 based on these randomizations were used to identify bound regions.

### Microarray expression data

RNA from mouse liver and cerebella was hybridized to Affymetrix Mouse Genome 430 2.0 arrays and analyzed as per the manufacturer's recommendations. Expression data was normalized using GCRMA [45]. Differential expression was assessed using Limma [46]. An adjusted p-value of 1e-3 was used to identify differentially expressed genes. Expression data for untreated, differentiated mouse 3T3-L1 cells was obtained from a previously published study [47]. Each probe set on the array is treated independently as a separate gene, and the array manufacturer's annotation data was used to obtain the TSS of the transcript targeted by the probe set. We note that the majority of the differentially expressed genes analyzed in this work mapped to a single probe set. In the liver/3T3-L1 analysis 1,818 genes were represented by a single probe set, while 120 were represented by more than one probe set. In the liver/cerebellum analysis 2,515 genes were represented by a single probe set and 384 were represented by multiple probe sets.

### Identification of nonconserved bound regions

We measured conservation levels in each bound region using Phastcons scores for 14-way alignments of placental mammals obtained from the UCSC Genome Browser. For each sequence we calculated a 100bp moving average of Phastcons scores and took the maximum observed value as the conservation score for that sequence. We then scored 20,000 sequences randomly selected from the mouse genome in an identical fashion. The conservation threshold of 0.35 was selected by determining the conservation level that best distinguished random sequences from bound sites in each dataset (Figure 6 in Text S1). Approximately 70% of random sequences fell below this threshold. We then identified a more stringent threshold, of 0.13, passed by only 35% of random sequences.

### Predictive model of expression from enhancer location

A transcript's expression rate is assumed to be a function of contributions from enhancers in the vicinity of the transcript's start site. The magnitude of an enhancer's effect on expression depends on its distance to the TSS. This is described by an influence function that we learn from the data. We also tested a similar model where, instead of fitting the influence function using binding position, we fit a curve using the conservation levels of binding events, allowing us to differentially weight regulatory regions with varying levels of sequence conservation. The model was further extended by allowing an enhancer's effect on expression to be modulated by the specific regulators bound through an influence weight, or by the affinity of a protein for the site (as measured by a ChIP enrichment ratio). These extensions are fully described in Text S2.

### Modeling absolute expression levels

Our goal is to predict log absolute expression level, as measured by a microarray experiment, using predicted enhancer locations. The rate of expression of a transcript, *k_1_*, is assumed to be a function of its basal expression rate, *k_0_*, and the action of nearby enhancers:
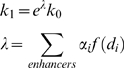
(1)Each enhancer is assumed to contribute additively to the expression rate modifier, *λ*. The effect that enhancer *i* has on this modifier is a function of its distance to the TSS, *d_i_*. It may also depend on other considerations, for example, the particular regulators bound at the enhancer. Such effects are subsumed into the parameter α*_i_*, which unless otherwise specified, is taken to be 1.

We assume 0^th^ order kinetics of mRNA production with rate constant *k_1_*, and 1^st^ order mRNA degradation kinetics with rate constant *k_2_*. We further assume that, measured across the population of cells, these processes are in equilibrium. The log transcript abundance is then given by:
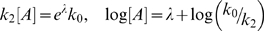
(2)The log intensity levels, *y*, from the Affymetrix arrays are noisy measurements of these transcript abundances. The mean squared error between the *N* observations and model predictions is given by:
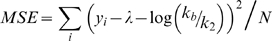
(3)We now express the enhancer influence function *f(d)* using a basis set of *P* 3^rd^ order B-splines [48]:
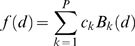
(4)Assuming that the term incorporating a transcript's basal expression rate and degradation rate, log(*k_0_*/*k_2_*), can be ignored leads to the following expression for MSE:
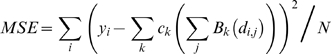
(5)The innermost sum over values of the B-spline basis functions for each enhancer position can be pre-computed. We introduce a penalty on an approximation to the integrated square of the 2^nd^ derivative of the fitted function to control complexity. The objective function we wish to minimize, *F*, then becomes:
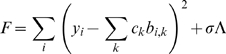
(6)Here *b_i,k_* are the pre-computed B-spline value sums over enhancers for basis function *k* and transcript *i*, *σ* is a regularization parameter that controls complexity, and Λ is the penalty term. The parameters defining the shape of the influence function, *c_k_*, can now be estimated by solving the system of equations:
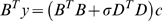
(7)where *D* is a matrix representation of the penalty term [48].

### Modeling relative expression levels

To predict relative expression levels between cell types *a* and *b*, we assume that basal expression rate and degradation rate for each transcript is identical in both cell types. The log fold change in expression, *y*, is then given by:
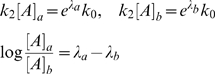
(8)and the mean-squared error is given by:
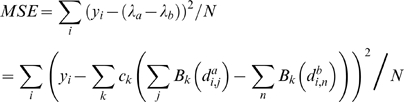
(9)Here enhancers present in tissue *a* are indexed by *j*, while those in tissue *b* are indexed by *n*. The influence function parameters are then solved as described above.

### Training and testing

For training, all genes with an enhancer within 100kb of the TSS were assembled. In each round of cross-validation, two thirds of these genes were randomly assigned to the training set, while one third were used for testing. Log expression values were mean-centered and normalized by the standard deviation. When training and validating models of differential expression between cell types, we limited our analysis to genes that were identified as being differentially expressed. The analysis of cerebellum and liver using ChIP-chip data used only enhancers located within the -5.5kb to 2.5kb region of promoters.

### Ethics statement

All experiments were carried out in accordance with guidelines for the use of laboratory animals and were approved by the MIT Institutional Animal Care and Use Committee.

## Supporting Information

Text S1 Supporting materials
(0.79 MB DOC)

Text S2 Supporting methods
(0.10 MB DOC)
