# Supplementary material for: A Quantitative Model of Transcriptional Regulation Reveals the Influence of Binding Location on Expression
Source: PLoS Comput Biol. 2010 Apr 29;6(4):e1000773. doi: 10.1371/journal.pcbi.1000773 (PMC2861697; doi:10.1371/journal.pcbi.1000773)
Supplement: Text S1 — Supporting materials (0.79 MB DOC) [file pcbi.1000773.s001.doc]

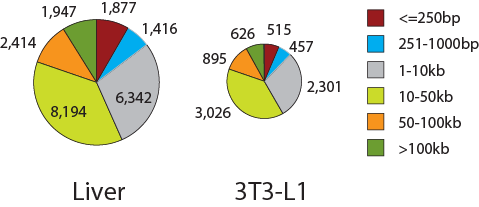


**Figure 1:** Distribution of proximities to the nearest transcription start site for regulatory regions identified in ChIP-seq experiments in liver and 3T3-L1 cells.


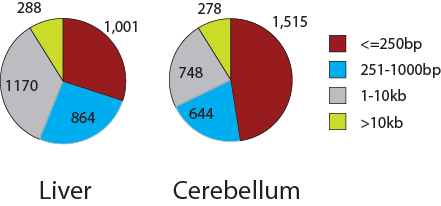


**Figure 2:** Distribution of proximities to the nearest transcription start site for regulatory regions identified in CBP ChIP-chip experiments in liver and cerebellum.


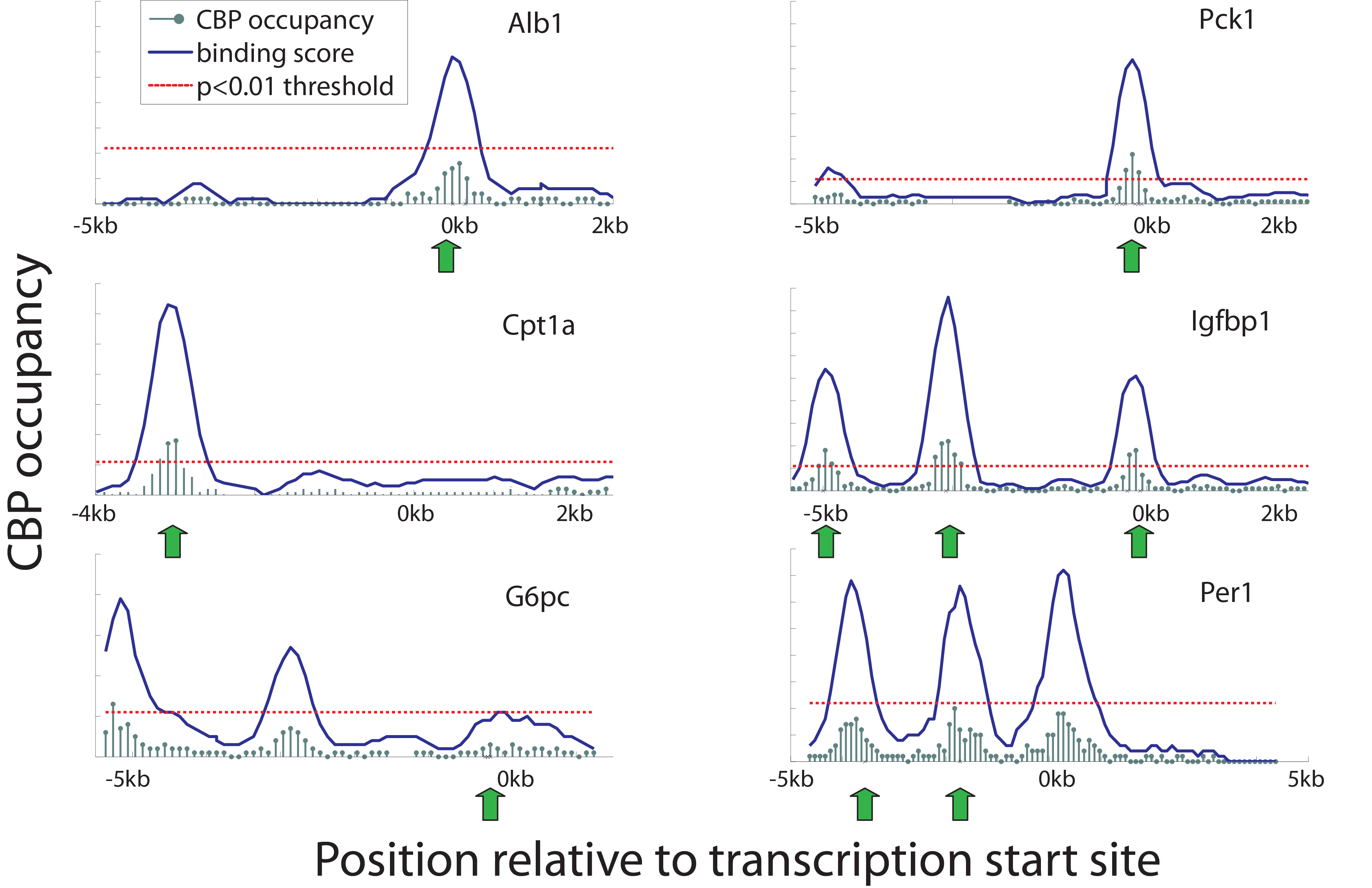


**Figure 3: CBP binding overlaps previously characterized enhancers.** CBP binding occupancy predicted by Redwing is shown in green, and smoothed CBP occupancy scores (obtained by convolving the Redwing predictions with a 400bp sliding window) are shown in blue. The red dashed line in each figure corresponds to a high confidence binding threshold (FDR <= 0.01). This threshold was estimated by running Redwing on randomly permuted ratio data. Positions of previously characterized enhancer regions are denoted by the green arrows. These include a proximal region between -170bp and the TATA box in the Alb1 promoter (Maire et al. 1989), a region at -3kb in the Cpt1 promoter (Louet et al. 2002), regulatory regions A and B between -231bp and ‑158bp in the glucose-6-phosphatase promoter (Onuma et al. 2009), a CRE and several other regulatory sites in the region -300bp to -100bp in the Pck1 promoter (Hanson and Reshef 1997), three distinct DNaseI hypersensitive regions at approximately -5kb, -3kb, and -300bp in the Igfbp1 promoter (Crissey et al. 1999), and characterized cAMP-response and glucocorticoid-response elements at -1728bp (Travnickova-Bendova et al. 2002) and -3566bp (Yamamoto et al. 2005) respectively.

**Figure 4: Expression level is related to regulatory site proximity in the proximal promoter.** Mean log expression intensity of transcripts from Affymetrix microarrays is plotted vs. the distance between their TSS and the nearest putative regulatory site in liver and 3T3-L1 cells. In this figure we restrict the analysis to genes with binding events in their proximal promoter.


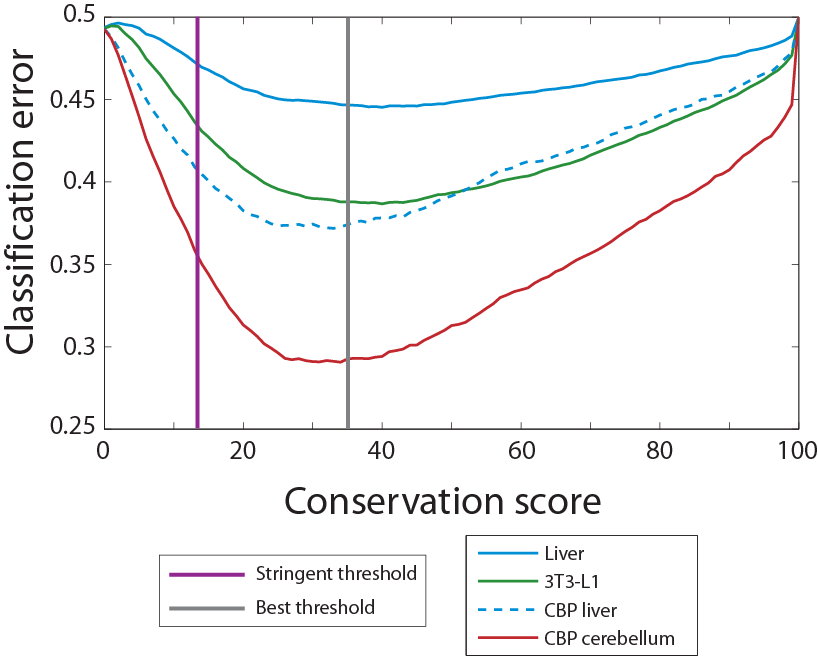


**Figure 5: Conservation thresholds.** We used conservation scores to distinguish bound regions in each tissue from sequences randomly selected from the mouse genome, evaluating classification error at 100 different thresholds. A conservation score of less than 35 was used to identify non-conserved sites since it yielded the lowest error rate across the four datasets. We identified a second, more stringent, threshold of 13 which yielded approximately 50% fewer non-conserved random sequences than the best threshold of 35.


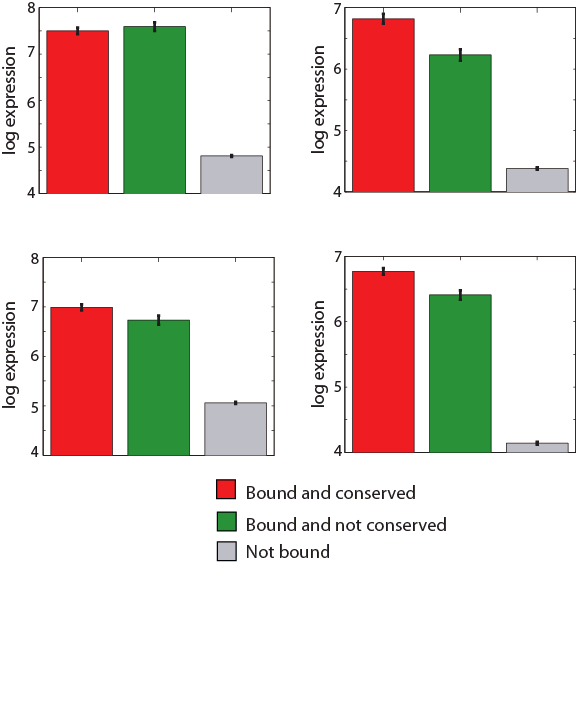


**Figure 6: Expression of genes bound at nonconserved sites.** Genes with only non-conserved regulatory sites within 5kb of their TSS have similar levels of expression to genes with conserved sites, and have a higher mean expression level than genes with no binding events. Conservation was identified using the best threshold shown above in figure S.5. Error bars indicate +/- s.e.m.

**Table 1 – Prediction of absolute expression level from enhancer position**

| **Experiment** | **MSE (random)** | **MSE** | **Correlation** |
| --- | --- | --- | --- |
| **CBP liver**  **(ChIP-chip)** | 1.007  +/-.003 | 0.915  +/-.003 | 0.30  (p=1.6E-32) |
| **CBP Cerebellum (ChIP-chip)** | 1.000  +/-.003 | 0.939  +/-.003 | 0.26  (p=3.3E-27) |
| **Liver**  **(ChIP-seq)** | 0.992  +/-.002 | 0.911  +/-.002 | 0.30  (p=1.0E-239) |
| **3T3-L1**  **(ChIP-seq)** | 1.000  +/-.002 | 0.939  +/-.002 | 0.239  (p=1.2E-127) |

Pearson correlation between absolute expression intensity measurements and model predictions on held out test data. The mean squared error and s.e.m. obtained by randomly guessing the training sample mean is shown in the first column. The mean squared error and s.e.m. of our model predictions is given in the 2^nd^ column, and their correlation with observed intensities (and associated p-value from a 2-tailed t test) is given in column 3.

**Table 2 – Cross-tissue conservation of binding sites near differentially and non-differentially expressed genes in liver and 3T3-L1 cells**

|  | **Differentially expressed** | | **No change** | |  |
| --- | --- | --- | --- | --- | --- |
| **Tissue** | **Total Sites** | **Unique** | **Total Sites** | **Unique** | **p-value** |
| **Liver** | 3,896 | 3,039 | 10,997 | 7,314 | 1.0e-42 |
| **3T3-L1** | 1,510 | 645 | 5,389 | 1,787 | 6.2e-12 |

All putative regulatory sites located within 10kb of differentially expressed genes and all sites located within 10kb of a non-changing gene (absolute value of log fold change<0.1) were identified in each tissue. Unique sites were defined as those that did not overlap a site in the second tissue. The reported p-value was calculated using Fisher’s exact test under the null hypothesis that the proportion of unique sites was the same for the ‘differentially expressed’ and ‘no change’ categories.

**Table 3 – Motif PSSMs derived from ChIP-seq and ChIP-chip data**

| **Data set** | **Literature consensus sequence (TRANSFAC)** | **PSSM derived from ChIP data** | **Z score** |
| --- | --- | --- | --- |
| **C/EBPa liver** | tt..gmAAt |  | 18.2 |
| **C/EBPa 3T3-L1** | tt..gmAAt |  | 9.8 |
| **E2F4 liver** | TTTsGCGC |  | 50.7 |
| **E2F4 3T3-L1** | TTTsGCGC |  | 43.2 |
| **FOXA2** | wraryAAAyA |  | 10.6 |
| **FOXA1** | .awTGTTTAtwT |  | 13.8 |
| **pCREB** | TGACGtma |  | 40.9 |

Sequence motif position-specific scoring matrices derived from ChIP data for the DNA-binding proteins profiled in this study. Motifs were learned from bound regions and randomly selected unbound DNA sequences using the previously published THEME algorithm (MacIsaac et al., *Bioinformatics* 2005).

**The association between binding site position and expression is not trivially explained by proximal promoter binding events.**

The enhancer influence functions and the empirical relationship between mean transcription level and the nearest enhancer location suggest that enhancers located <10kb from the TSS are particularly crucial for predicting expression level. In order to probe whether position is important in predicting expression even within this relatively narrow window, and to demonstrate that the predictive power of our modeling framework is not trivially explained by differences in binding at the proximal promoter, we excluded from our analysis all enhancer regions located greater than 10kb or closer than 1kb to a TSS and repeated model training and testing. In liver and cerebellum, this left 1665 differentially expressed genes while in liver and 3T3-L1 cells we were left with 1040 differentially expressed genes. For both data sets, the predictive relationship between enhancer location and differential expression remains strong. Furthermore, when we compare predictive performance to a simpler model that ignores enhancer location and predicts differential expression based solely on changes in the number of putative enhancers within 10kb of the TSS, we find that position is clearly important in predicting the effect an enhancer’s presence has on expression level.


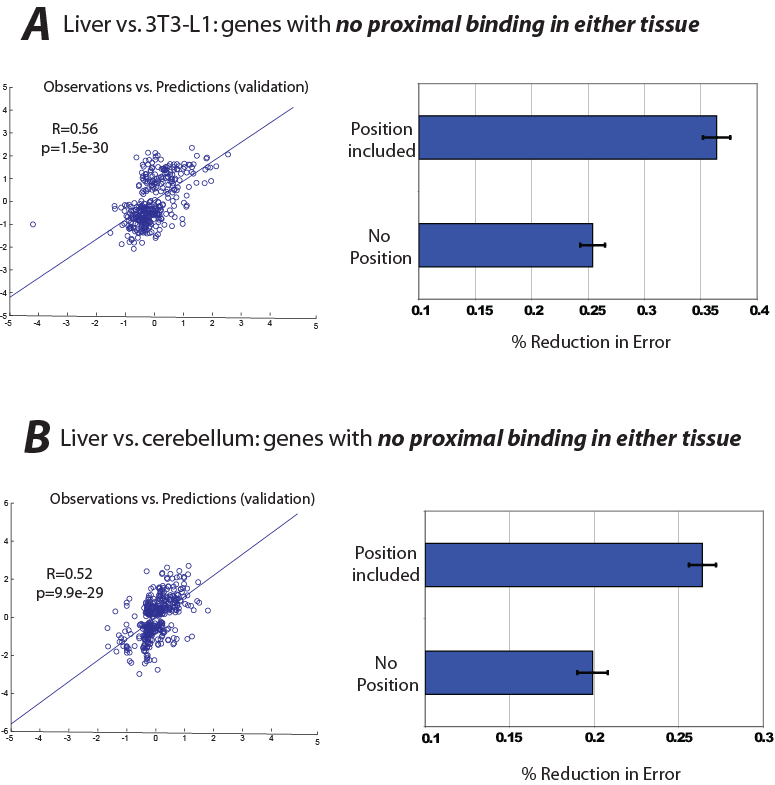


**Figure 7: Enhancer position predicts expression for genes with no proximal binding events.** Representative scatter plots of observed and predicted normalized expression difference for held-out validation data are presented for differentially expressed genes in liver and cerebellum (A) and genes in liver and 3T3-L1 cells (B) with no binding events within 1kb of the TSS. To demonstrate the importance of position even for binding events within 10kb of regulated genes we excluded any binding event located more than 10kb from the gene’s TSS. Median correlation between observed and predicted expression difference is greater than 0.5 and highly statistically significant for both analyses. The reduction in mean test error relative to random guessing is shown for the full predictive model and for a model that ignores binding position. In both cases, modeling the effect of position significantly improves performance.


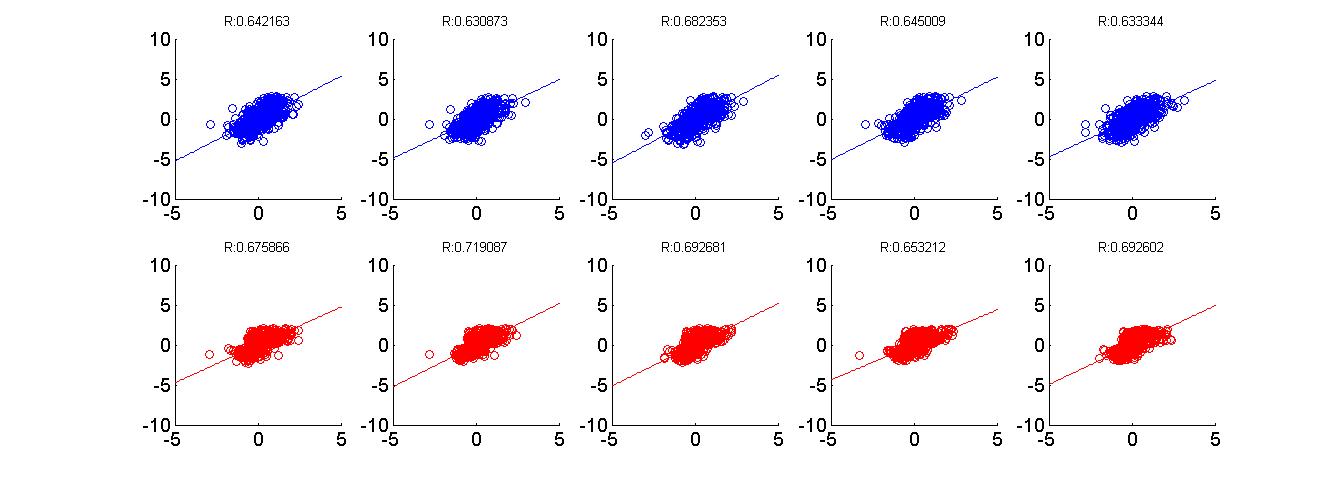


**Figure 8: The correlation between log fold expression difference measurements and predictions is robust.** Representative scatter plots of observed expression difference (y-axes) and predicted expression difference (x-axes) for held out test data are shown for the liver and cerebellum CBP data (red) and the liver and 3T3-L1 combined ChIP-seq data (blue). In each case 2/3 of the data was randomly sampled and used to train the predictive model while the remaining 1/3 was held out and used for testing. Correlation coefficients (R) between observations and predictions are shown for each bootstrap trial.
