# Supplementary material for: A Quantitative Model of Transcriptional Regulation Reveals the Influence of Binding Location on Expression
Source: PLoS Comput Biol. 2010 Apr 29;6(4):e1000773. doi: 10.1371/journal.pcbi.1000773 (PMC2861697; doi:10.1371/journal.pcbi.1000773)
Supplement: Text S2 — Supporting methods (0.10 MB DOC) [file pcbi.1000773.s002.doc]

**Supplemental methods**

***1 Extensions to the transcriptional regulation model***

***1.1 Modeling the effect of regulators bound at the enhancer***

When data for the binding of several regulators at enhancer regions is available, we can model their individual effects on enhancer function by introducing a parameter θ_m_ for each regulator that modifies an enhancer’s effect on transcription (equation 1 in the methods section) as follows:

$\begin{matrix} \lambda=\sum_{enhancers} \alpha_{i}f\left(d_{i}\right) \\ \alpha_{i}=\prod_{m} \theta_{m}^{I_{i,m}} \end{matrix}$ (1.1)

Here *I_i,m_* is an indicator variable taking the value of 1 if regulator *m* is present at enhancer *i*, and 0 otherwise. The objective function we wish to minimize is then given by:

$F=\sum_{i} \left(y_{i}-\sum_{k} c_{k}\sum_{j} \left[\left(\prod_{m} \theta_{m}^{I_{j,m}}\right)B_{k}\left(d_{i,j}\right)\right]\right)^{2}+\sigma\Lambda$ (1.2)

Taking the derivative with respect to *c_k_* yields:

$\frac{\partial F}{\partial c_{p}}=-2\sum_{i} \left\{\left(y_{i}-\sum_{k} c_{k}\sum_{j} \left[\left(\prod_{m} \theta_{m}^{I_{j,m}}\right)B_{k}\left(d_{i,j}\right)\right]\right)\left(\sum_{j} \left[\left(\prod_{m} \theta_{m}^{I_{j,m}}\right)B_{p}\left(d_{i,j}\right)\right]\right)\right\}$ (1.3)

Taking the derivative with respect to θ_m_ yields:

$\frac{\partial F}{\partial\theta_{r}}=-2\sum_{i} \left\{\left(y_{i}-\sum_{k} c_{k}\sum_{j} \left[\left(\prod_{m} \theta_{m}^{I_{j,m}}\right)B_{k}\left(d_{i,j}\right)\right]\right)\left(\sum_{k} c_{k}\sum_{j} \left[I_{j,r}\left(\prod_{m\neq r} \theta_{m}^{I_{j,m}}\right)B_{k}\left(d_{i,j}\right)\right]\right)\right\}$ (1.4)

We then set these derivatives to zero and solve the resulting system of equations using the nonlinear equation solver fsolve in Matlab to obtain parameter estimates for the c’s and θ’s.

***1.2 Modeling the contribution of binding affinity***

For the CBP binding data, we model the contribution of binding affinity to expression influence by including a sigmoidal weighting term ψ*(x)* to modify λ in equation 1 from the Methods:

$\begin{matrix} \lambda=\sum_{enhancers} \psi\left(x_{i}\right)f\left(d_{i}\right) \\ \psi\left(x\right)=\frac{1}{1+\exp\left(\mu-x\right)} \end{matrix}$ (1.5)

Here *x* is a measure of binding enrichment at the regulatory site, in this case the mean-subtracted, standard deviation normalized ChIP enrichment ratios from a ChIP-chip experiment. The parameter *μ* is selected using an internal round of cross-validation to optimize the accuracy of expression prediction. We selected *μ* from the values: -1e6, -3, -2.5, -2 … 2.5, 3, and 1e6. This allowed the algorithm to weight the ChIP enrichment ratio of a site very highly, an intermediate amount, or not at all.

***2 The Redwing algorithm for analyzing ChIP-chip data***

The Redwing algorithm uses ChIP-chip probe ratio information to infer the binding location and occupancy of a protein in a genomic region. Our approach is motivated by a simple physical model of how protein associates with DNA *in vivo* and how these associations are observed as noisy probe ratio measurements in a ChIP-chip experiment. In a ChIP-chip experiment, we detect the presence or absence of a protein of interest at arrayed regions in the genome. Consider a single binding site for the protein: in some cells in the population the protein will be present and cross-linked to the DNA, while in others the protein will not be present. Only sites in the population that have a cross-linked instance of the protein will contribute to the intensity measurement in the experiment. We define the occupancy of a particular genomic region as the expected number of protein molecules bound in that region, per cell. Redbird assumes that the intensity ratios of probes in the vicinity of a binding site are related to the occupancy in a manner that depends on the distance between the site and the probe location.

***2.1 Protein-DNA association***

Redbird models the recruitment of a protein, *A*, to a region of DNA, *B*, as a bimolecular reaction at equilibrium, leading to the formulation for binding probability given in equation 2.1:

$p=\frac{1}{1+\exp\left(-\beta_{0}+\sum_{i} \beta_{i}x_{i}\right)}$ (2.1)

Here, the *β_0_* parameter is the log of the free protein concentration. In general, the *x_i_* can be used to incorporate sequence motif features that may affect binding affinity, however in this study we ignore these effects and assume affinity is the same everywhere in the genome. Binding probability is then given simply by:

$p=\frac{1}{1+\exp\left(-\beta\right)}$ (2.2)

***2.2 Modeling ChIP probe ratios***

The probe ratio data is assumed to be a noisy representation of a protein’s binding occupancy averaged across *N* cells. We model the observed probe ratios as the sum of a signal and noise term:

$r=s+\varepsilon$ (2.3)

The noise term, *ε*, accounts for the distribution of probe ratios observed when no binding occurs in a probe’s vicinity and is distributed $\mathrm{Gamma}\left(\frac{1}{\theta},\theta\right)$. The signal term, *s*, is assumed to be proportional to the occupancy of the immunoprecipitated protein at the locus tiled by the probe. Consider a binding site where in *n* cells the protein is cross-linked to the DNA. Each of the *i*=*1*…*n* binding events is assumed to contribute to the total measured signal through the gamma random variables *b_i_*, having shape and scale parameters *k* and *θ*. *s* is the sum of these contributions:

$s=\sum_{i=1}^{n} b_{i} where b_{i}\sim Gamma\left(k,\theta\right)$ (2.4)

Since the observed probe ratio is the sum of *n* gamma random variables with identical scale parameter, it is itself a gamma random variable distributed $\mathrm{Gamma}\left(nk+\frac{1}{\theta},\theta\right)$.

After cross-linking in a ChIP experiment, the DNA is fragmented prior to immunoprecipitation. Depending on the size distribution of this fragmented DNA, binding events located hundreds of bp away from a probe can have an appreciable effect on that probe’s observed ratio value. Following Qi et al.^44^, we seek to deconvolve the tiled ratio data to identify binding locations most consistent with probe ratio values and the expected shear distribution of the immunoprecipitated DNA. We account for the effect that nearby binding events have on a probe’s observed ratio using an influence function, $g\left(d\right)$,that models how this effect decays with increasing distance, *d*, between binding site and probe. Here, the influence function is just a scaled Gaussian with a standard deviation of 250bp, and a maximum value of 1.0 centered on the binding event. This is approximately consistent with the distance decay of the influence functions calculated from experimentally measured shear distributions^44^. The effect of the influence function is to attenuate the contribution of individual binding events located distal to the probe location. For *i*=*1*…*n* binding events located at distances *d_i_*=*1*…*n* from the probe, their individual contributions, *x_i_*, to the total signal, *r*, are given by:

$\begin{matrix} r=\varepsilon+\sum_{i=1}^{n} x_{i}, x_{i}\sim Gamma\left(kf\left(d_{i}\right),\theta\right) \\ r\sim Gamma\left(\frac{1}{\theta}+k\sum_{i} g\left(d_{i}\right),\theta\right) \end{matrix}$ (2.5)

***2.3 Unified Model***

The experimental data consist of probe ratio values from the *P* spots on the microarray(s) (indexed by *i=1…P*). Independent replicates of each experiment have been performed (indexed by *j=1…R*). The probabilities of each observed probe ratio value, *r_i,j_*, are assumed to be conditionally independent given the binding occupancies of the immunoprecipitated protein at each location in the genome. We discretize the binding occupancies by modeling the whole population of cells from the ChIP experiment using *N* virtual copies of each region on the array. Here we set *N*=100. We assume that the probe ratio probabilities are independent given the occupancy data. The likelihood of the probe ratio data is then simply the product of *PR* gamma pdfs:

$\begin{matrix} L\left(data\right)=\prod_{i=1}^{P} \prod_{j=1}^{R} r_{i,j}^{k_{i}-1}\frac{e^{-r_{i,j}/\theta}}{\theta^{k_{i}}\Gamma\left(k_{i}\right)} \\ where k_{i}=k\sum_{n=1}^{N} \sum_{m=1}^{M} b_{n}g\left(d\left(i,m\right)\right) \end{matrix}$ (2.6)

As discussed above, the shape parameter *k_i_* for each probe depends on both the occupancy and distance of nearby binding events. The term *b_n_* is an indicator variable that is set to one if virtual copy *n* of region *m* is bound, and is zero otherwise. The more virtual copies bound (i.e. higher occupancy) the larger the scale parameter. The effect of each virtual binding event is attenuated by the influence function *g*(*d*(*i*,*m*)) whose value depends on the distance, *d*(*i*,*m*), between probe *i* and region *m* as discussed above.

***2.4 Learning procedure***

The Redbird algorithm uses a Monte-Carlo EM procedure to infer binding locations and occupancies, and to learn the various parameters of the model. After parameter initialization, the optimization proceeds by first sampling an ensemble of binding configurations for each set of *N* virtual regions. Then, given these samples, we update the parameter values to maximize the expected likelihood of the data. The parameter *κ*, which determines the average occupancy of any genomic region, is initialized so that a random 100bp window will have an average occupancy of 1 molecule per 100 cells. The probe ratio distribution parameters *θ* and *k* are initialized to 0.1 and 0.4 respectively. The mean ratio of an unbound probe is therefore 1.0, and the mean ratio of a probe occupied at a level of 100 molecules of protein/cell is 5.0.

***2.4.1 E-step***

In the E step we wish to obtain expected binding occupancies given the probe ratio data in each genomic region. We employ a sampling method, inspired by the Gillespie stochastic simulation algorithm^1^, which can be derived by viewing each genomic region as a chemical system at equilibrium. Imagine we have a well-mixed reaction vessel where *i*=1…*N* different reactions can occur. The time of the next reaction of type *i* is distributed exponentially:

$\begin{matrix} P\left(\tau\right)=h_{i}c_{i}\exp\left(-h_{i}c_{i}\tau\right) \\ =a_{i}\exp\left(-a_{i}\tau\right) \end{matrix}$ (2.7)

Where *h_i_* is the number of distinct combinations of reactants that can combine according to reaction *i*, and *c_i_* is the rate constant. Assuming constant chemical potentials, each reaction is an independent Poisson arrival process and therefore the time of the next reaction of any type is also a Poisson process with inter-arrival times distributed exponentially:

$P\left(\tau\right)=\sum_{i=1}^{N} a_{i}\exp\left(-\sum_{i=1}^{N} a_{i}\tau\right)$ (2.8)

We can view the unfolding of a reaction time course as a Poisson splitting process with inter-arrival times distributed according to (2.8). Each arrival is sent to an individual reaction channel, *i*, with probability:

$p_{i}=\frac{a_{i}}{\sum_{j=1}^{N} a_{j}}$ (2.9)

The execution of a reaction changes the chemical potential for other reactions so this Poisson process is non-homogeneous. However, by sequentially drawing samples from these two distributions and updating the *a_i_*’s we arrive at the well-known Gillespie stochastic simulation algorithm (SSA) for exactly sampling the trajectory of a reacting system.

Applying a modification of the SSA, we obtain samples from an approximation to the posterior equilibrium distribution of binding events. The reactions that must be modeled are simply protein association and dissociation from DNA, however in general this scheme could be expanded to include other reactions like dimerization of proteins to form a bound complex. The original Gillespie algorithm assumes that all relevant rate parameters are known and then proceeds by iteratively selecting the next reaction based on these rate constants and the current number of reactant molecules (i.e. the chemical potential). To obtain the rate constant for a reaction we make two observations: the relative rates of the forward and reverse reactions at equilibrium are given by the parameter β in equation (2.2), and secondly, since we are concerned only with the equilibrium behavior of the system rather than its time-course kinetics, these relative rates are all that is required in our simulation as long as sampling is run for a time course long enough to achieve equilibrium.

We incorporate posterior binding evidence by calculating an effective chemical potential for the reaction. Consider the case where we have evidence, *X*, regarding the equilibrium state of the system. We wish to bias the reaction time course so that reactions more consistent with the evidence are favored. We accomplish this by altering the Poisson splitting process. Rather than splitting the arrivals according to (2.9), we split according to the posterior probability of a reaction given the evidence:

$\begin{matrix} P\left(r_{i}|X\right)=\frac{P\left(X|r_{i}\right)P\left(r_{i}\right)}{\sum_{j=1}^{N} P\left(X|r_{j}\right)P\left(r_{j}\right)} \\ =\frac{P\left(X|r_{i}\right)a_{i}}{\sum_{j=1}^{N} P\left(X|r_{j}\right)a_{j}} \end{matrix}$ (2.10)

Near the posterior equilibrium point the probability of the evidence given that the next reaction is *r_i_* will be approximately equal for each reaction, (2.10) will be approximately equivalent to (2.9), and we will approach the standard SSA algorithm. This sampling procedure is run for a reaction time selected to produce on average 50 total on/off reactions per probe, a reasonable number since most probes tile unbound regions and will therefore use up very few simulated reactions relative to probes with strong evidence of enrichment. At the end of sampling, the total occupancy across 100 virtual copies of each binding site is interpreted as *E[occupancy]*.

***2.4.2 M-step***

We use the binding occupancy estimates obtained in the E-step to update the various parameter estimates such that the likelihood of the data improves after each iteration. Optimization of *k* and *θ* is performed using the Nelder-Mead simplex method implemented as a standard package in Matlab. This search method requires only that one be able to calculate the likelihood function. When performing optimization we calculate the likelihood function given in (2.6) but add in a penalty term to bias the optimization towards values of *k* and *θ* that yield a mean probe ratio of 5.0 when a probe is occupied at a level of 1 molecule/cell (i.e. 100%) as follows:

$$\begin{matrix} l\left(data\right)=\sum_{i=1}^{P} \sum_{j=1}^{R} \left[\left(k_{i}-1\right)\log r_{i,j}-\frac{r_{i,j}}{\theta}-k_{i}\log\theta-\log\Gamma\left(k_{i}\right)\right]-a\left(100k\theta-4\right)^{2} \\ where k_{i}=k\sum_{n=1}^{N} \sum_{m=1}^{M} b_{n}f\left(d\left(i,m\right)\right) \end{matrix}$$

The parameter *a*, is set to 1e5; this is roughly equivalent to placing a Gaussian prior with mean 5 and standard deviation of 2.2e-3 on the ratio value of a fully occupied probe.

The log of *β* is equal to the log-odds of binding, therefore the prior probability of the observed binding given *β*, is simply the product of logistic functions:

$$P\left(B\right)=\prod_{m=1}^{M} \prod_{n=1}^{N} \left(\frac{\exp\left(-\beta\right)}{1+\exp\left(-\beta\right)}\right)^{b_{m,n}}\left(\frac{1}{1+\exp\left(-\beta\right)}\right)^{1-b_{m,n}}$$

Taking the derivative of the above equation with respect to *β* and setting it equal to zero gives:

$$\left(\frac{\exp\left(-\beta\right)}{1+\exp\left(-\beta\right)}\right)=\frac{1}{MN}\sum_{m=1}^{M} \sum_{n=1}^{N} b_{m,n}$$

Solving this equation numerically yields our updated estimate of *β*.

**1. Gillespie, D.T. Exact Stochastic Simulation of Coupled Chemical-Reactions. *Journal of Physical Chemistry* 81, 2340-2361 (1977).**
